# Observations on the New Medicine Act

**Published:** 1802-08-01

**Authors:** 


### *Observations on the New Medicine Act; communicated by Mr.* Chamberlaine.

Medicus es. Nihil ad medicinam pertinens a te alienum esse puto.

A Most alarming scourge hangs over the head of every Medical Man, practising Pharmacy, whether publicly or privately, after the first of September next.

The evils under which the practitioner in Pharmacy labours, are already too great: it was needless to add to the burthens of those who are already so much harrassed by difficulties.

Some years ago a bill was brought into Parliament, which the honourable mover stated was for the protection of the Regular Practitioner, by imposing a duty on Quack Medicines, and obliging the Proprietors, Patentees, and Vendors of them to take out licences. The Act, in its original plan, might have been well intended; but through the inaccuracy of those employed to draw it up, the guard became the assailant.

For, while the *avowed empiric* was safe, the loose wording, and inexplicable clauses of the act, rendered various honest and industrious men subject to the penalties, who never intended to offend against the act, but who, from never having any thing to do with nostrums, supposed they could not offend against an act of the Legislature, apparently applying to nostrums only.

Every good subject must allow the necessity there is, that the government of a country should be supported; and that the security which its inhabitants enjoy, must be paid for by duties and imposts laid on the subject.

Some of these imposts bear hard enough on certain classes of the community, and on private individuals. We must, however, submit to them, when they cannot be ameliorated; but when they tend to the absolute ruin of honest and unintentionally offending men, then it becomes our duty to look to ourselves, collectively as well as individually, and unite in firm but legal and moderate opposition, to a measure pointedly directed against that particular body of men of which we compose a part; and fraught with ruin that cannot possibly be averted, even by the most cautious, from falling on us, and bringing to poverty, to prisons, and work-houses, ourselves, our wives, our children, and all that is dear to us.

Of such a complexion is a certain act of the late Parliament, passed the 3d of June, purporting to be "An Act to repeal peal the former Quack Medicine Act, and for making effectual Provifion for the better Collection of the new Duties "

If the former act was perplexing, this is more fo. In the 19th claufe (a moft tremendous claufe) it is ftated, that certain doubts are to be removed; but it only explains ignotum per ignotius; and if it is intended to be an amendment of the old act—it is an amendment with a vengeance!

Medical Gentlemen practifing Pharmacy, but not keeping retail fhops, nor having licences to vend empirical noftrums, may think that this act will not affect them; but they will find themfelves grievoufly miftaken: it concerns every Apothecary in England, either in private practice or keeping a public fhop.

In a Schedule, confifting of *twelve folio columns*, and comprehending nearly fix hundred different articles, we find the following, which are only a few, felected out of a great many others, equally harmlefs, and not at all allied to quackery.

Turkey rhubarb.
Indian arrow-root.
Huxham's tincture of bark.
Syrup of tolu.
Bliftering ointment.
Spanifh juice.
Refined liquorice.
Healing falve for burns and fcalds.
Nitre Drops, (i. e. fp. ætheris nitrofi.)
Goulard's extract.
Kibe ointment.
Lozenges of all forts.
Lip-falves of all forts.
Iffue-plafters of all forts.
Tooth-powders *of all forts.*
Ophthalmic lotions and collyriums.
Camphorated eye water.
Sedative and ftrengthening eye-water.
Red Pills (i. e. any pills rolled in bole armenic, or vermillion.)
Chalybeate pills. Nervous pills.
Quaffia pills. Tooth-ach pills. Tonic pills.
Candied horehound and candied ginger.

How fome of thefe, or indeed any of them, were deferving the badge of empiricifm, no one can tell but the framer of the bill.

What harm Indian arrow-root had done, or why tapioca fhould efcape, is as difficult to be afcertained, as why fyrup of tolu fhould be felected from the whole fet of fyrups of the Difpenfatory, and no notice be taken of the fyrups of faffron, mulberries, mulberries, or blackthorn! Why common Spanifh juice fhould be laid hold on, and *Turkey* rhubarb fold with a ftamp, while any rhubarb but Turkey rhubarb may go free, is a myftery I muft leave to wifer heads to develope; nor can I reconcile it to my ideas of juftice, that any man fhould be punifhed by a fine of £. 30 for felling a man a plafter for a fcalded foot!

In many parts of the Schedule, the reader meets with names that never had any exiftence, and others which are downright nonfenfe.

For inftance, we have balfam of *Ire*land liverwort, (inftead of *Ice*land) Anti*petussus*, and *Anterticumatic* drops. Hewitt's *Arralambarric* pills; and for fear this fhould not be right, we have, a little lower down, Hewitt's *Analambannic* pills. Effence of *Kayon Ponti*, and *Tuberoga* vitæ. Wherever the word Aperient ought to have occured, it is uniformly printed A*s*perient. This might be excufed as an error of the prefs; but truly, the compiler of this bleffed Schedule feems to have been pretty *sedulous* not to make miftakes; and as a proof of this care, we have in one place Gowland's *celebrated* lotion; but for fear there fhould be *another* lotion of Gowland's *without* any celebrity, he inferts, lower down, fimply, Gowland's Lotion. In the 9th column of this Schedule, we find, fedative collyrium, ftrengthening ditto; but, to *remove doubts*, in the tenth column, we have it again with improvements, fedative Collyrium for inflamed eyes, and ftrengthening Collyrium for weak eyes!

The former act of parliament, impofing a duty on Quack Medicines, made in the 25th Geo. III. applied only to fuch as were kept fecret, or generaly held to be fecrets, in the hands of particular perfons; to Patent, and Proprietary Medicines; but it is a matter of the utmoft importance to take notice, that by the 19th claufe of the new act, every medicine, whether kept fecret or not, whether fold by regular or quack, that is fpecified in the Schedule, muft have a ftamp, and the feller a licence: and this act extends not only to every article fet forth in the Schedule, noftrum or not noftrum, but to all compofitions, mixtures, powders, &c. &c. "*which at any time heretofore have been, now are, or hereafter fhall be, by any public notice or advertisement, or by* WRITTEN *or printed papers or hand-bills, or by any labels or words, written, printed, or affixed to, or delivered, with any such packet, box, bottle, phial, &c. recommending it* (even without claiming any fecret mode of preparation, or proprietary right in vending the fame) *as beneficial for the prevention, cure, or relief of any distemper, ailment, malady, or complaint incidental to the human body.*"——See claufe 19.

Now, Sir, by this it appears, that no Medical Gentleman who practiſes Pharmacy can follow his profeſſion in ſafety, becauſe, ſeeing that by this ſweeping NINETEENTH Clauſe, it is enacted, that any label, printed or written, or any direction ſetting forth *in any wiſe* the QUALITY of the medicine or the COMPLAINT it is to relieve, ſhall ſubject the perſon who ſells, or utters the ſame, to a penalty of ten pounds for ſelling the medicine without a ſtamp; and if the ſeller has no licence, to the further penalty of twenty pounds in addition thereto. So it follows, that if any gentleman affixes a common label, with the words, " *The pectoral mixture, a ſpoonful to be taken when the cough is troubleſome,*" or writes " *Purging pills* " on the lid of a box, he is, in my humble acceptation of the meaning of the act, liable to the penalty.

Now, you gentlemen who have your ſhops in your back parlours, or your private Pharmacothecæ in your cloſets, on the ſecond floor, who hug yourſelves under the impreſſion that this act was made for us poor fellows that keep retail ſhops, and that as YOU keep no retail ſhops, nor vend noſtrums, the act has nothing to do with you; are you, think you, out of its reach? By no means. The informer will find ways and means to ferret you out of your ſecure holes, and raiſe contributions on you as well as the Retailers, who certainly are *much more* open to their depredations, and whoſe RUIN, unleſs this act is repealed, muſt be INEVITABLE.

Let us now look to the operation of this dangerous act, and I will ſuppoſe a caſe of a gentleman, who keeps no retail ſhop, and thinks himſelf pretty well out of the reach of informers.

We will ſuppoſe that one of the gang of the informers whom this act will let looſe upon the medical tribe, on the grocers, the confectioners, has it in contemplation to lay all theſe deſcriptions of perſons, reſiding in a particular diſtrict that he fixes on, under contribution.

The informer begins his plan of operations, by taking genteel lodgings, and perhaps, may take his wife and child with him. He ſends for the Surgeon or Apothecary, relates an account of ſham complaints; medicines are ſent in, which he puts in the fire, and next day, when the Doctor comes, he expreſſes his thanks, is nearly recovered, and puts the ſon of Æſculapius in a good humour with him, and with himſelf alſo. He will continue his medicines one day longer; " and then, Sir," ſays he, "you will do me the favour to ſend in your bill."

Next day the bill is paid, and perhaps a compliment over; juſt as Galen is making his bow, his patient recollects that he is ſometimes troubled with a bowel complaint, for which he finds nothing ſo good as a little Turkey rhubarb, which he is in the the habit of chewing. Send me an ounce. "Yes, Sir." The lady wife, (and let me tell you, Sir, the *Ladies* employed in the bufinefs of informations are much more numerous and more wily and infidious than the men;) the lady then comes on in *her* part. "Oh! Doctor, and when you fend the rhubarb, pray fend me fome fyrup of tolu for my child's cough." The order is executed, and next day the practitioner receives a fummons to appear before a magiftrate; he is convicted in the penalty of ten pounds for felling an ounce of Turkey rhubarb without a ftamp, ten pounds more for his phial of fyrup of tolu, and twenty pounds for felling them not being duly licenfed. The magiftrate mitigates it to one half, but cannot reduce it lower; fo, here is a gentleman robbed of twenty pounds (or more, of his time and his reputation, through inadvertence, or perhaps through ignorance that fo fevere a law could be tolerated in England!

But let us now turn to the fituation of the poor, hard working, indigent Apothecary, who, by a little retail at the outfkirts of fome town, fcarcely earns enough to fupport a wife and five or fix fmall children, and forry am I to fay it, many fuch there are. We will fuppofe fuch a one as I have here defcribed, whofe clear earnings may not, perhaps, amount to fo much as £. 150 per annum, marked as the prey of fome of thefe depredators, who go about feeking whom they may devour.

We will firft fuppofe a very poffible cafe; namely, that this man has read the Act of Parliment, and is on his guard, and that a perfon comes in with a bad fcald on his foot, and afks for a pennyworth of "Healing falve for a fcald." The apothecary anfwers, "I cannot fell you any healing falve for your fcald without you take a three-halfpenny ftamp with it. The ftranger anfwers, "I have but one penny in the world." What is to be done? If he gives it away as an act of charity, he runs a hazard; and even if he applies a dreffing gratuitoufly, he is equally in danger. The fufferer muft endure his pain, and go away unrelieved, leaving him who was both able and willing to adminifter eafe, to lament that a rigorous act of parliament precluded him from extending an act of humanity to a fuffering fellow creature, without endangering the lofs of great part of his property.*

* The facility with which informers can entrap thofe who are without apprentices or fhopmen, and where there is no one but the hufband or wife to ferve, needs no comment. The want of a witnefs to confront the evidence given by the informer's witnefs, leaves the defendant without means of defence, and without hope of redrefs!

But we will now fuppofe an equally poffible, and ftill more probable cafe; namely, that while an apothecary, fuch as I have defcribed, is from home, a ftranger applies at his fhop for the fame article. Too poor to keep an apprentice, or to afford to have a journeyman, the wife is the only one to ferve in the fhop. She, who has befides, the work of the houfe to do, and four or five children to attend to, cannot be fuppofed to be deeply verfed in acts of parliament. She fells her cuftomer that which fhe fuppofes the beft falve to heal a burn or fcald, and thinks no harm of it.

Prefently after comes in a well-dreffed woman for fome nitre drops; the wife unfufpectingly ferves her with a little fpir, ætheris nitrofi.

A third perfon comes in with a fore mouth, "I will thank you, miftrefs, for fome fort of falve to heal this lip of mine." The good woman thinks no harm in ferving her cuftomer with a pennyworth of fpermaceti ointment for his fore lip, and takes the money.

Next day, the diftrefs of the family, on the mafter of the fhop being ferved with a fummons to appear before a Magif-rate, on three feparate informations; the one for felling *healing salve for a scald*, the fecond for felling *nitre drops*, and the third for felling *lip-salve*, without ftamps, is not to be defcribed.

The Magiftrate, on hearing the merits of the cafe, will, if he is a gentleman, and a humane and confcientious man,* exclaim, "God blefs my foul, I cannot think the Legiflature ever meant to tax things fold in this manner! Such names are in the Schedule to be fure; but I rather think they apply to articles of fimilar nature, kept fecret in the hands of fome particular perfon, and fold as noftrums. I cannot ruin this poor man by a conviction on fuch vague and uncertain ground."

The informer, who perhaps has his attorney at his elbow, will reply to the Magiftrate, "Sir, you are not to interpret the

* The eftablifhment of Police Offices throughout the metropolis, where none but gentlemen of refpectability are appointed to prefide, ftruck the death-blow to the occupation of a number of needy people, who kept Juftice-Shops, or rather INjuftice-Shops, in various parts of the town; and had no other way of earning a livelihood, than by convicting as many as they could, and living by the earnings of iniquity. At the different Police Offices, the worthy magiftrates, who there adminifter juftice, (to do *them* juftice) fet their faces againft thefe vexatious informations as much as, confiftently with their duty, they dare do; but although the metropolis is purged of trading juftices, are we fure that there do not exift, in many of the towns and villages throughout the kingdom, men in the commiffion of the peace, who, through ignorance, fear, or fome other motive, would be as ready to convict as the trading juftices of London formerly were?

the act of parliament as you pleafe, nor find meanings in it to fuit your own purpofe. Read the nineteenth claufe, and there you will find that claufe exprefsly fays, that the rates impofed by the act fhall extend to all and every the articles mentioned in the Schedule. Read the *Schedule*, and you will *there* find "*healing salves for burns or scalds*, nitre drops," and "*lip-salves of all sorts.*" "The Schedule does not name thefe things as being the *property*, or going under the *name* of any particular perfon; it applies *generally*; therefore, if you refufe the conviction, I fhall appeal to the quarter feffions."*

Taking it for granted that the poor man muft be convicted, here is, "at one fell fwoop," thirty pounds penalty upon three convictions, of ten pounds each, which the Magiftrate, in compaffion to the innocent man and his family, mitigates to one half, lamenting that the rigour of the law will not allow him to remit ftill more. With a heavy heart, the ill-fated fon of Galen is departing the office, pondering how he fhall raife the fifteen pounds; but we have not done with him yet: he is called back, and told there is *another* information remaining againft him, *viz.* for felling the articles on which he has been convicted, WITHOUT *a* LICENCE. The penalty for this offence is twenty pounds, which the Magiftrate mitigates to ten; and there is alfo fomething more to be paid for the fees of office!—So, here is what would cloath and educate two or three children for a whole year, facrificed, in order that a few unprincipled wretches may get a few fhillings! †.

A few more fuch fevere blows as thefe would inevitably fend fuch a man as I have defcribed, and his family, to the workhoufe.

Gentlemen

---

* An appeal to the quarter feffions is attended with very confiderable expence to the defendant; not lefs than £.15 or £.20. As the qui-tam action is brought by the informer, tam pro rege, quam pro fe; and as it is a maxim in law that the King pays no cofts, the defendant, if cleared, is faddled with the whole of the cofts; and if convicted, he has the penalties to pay befides thefe cofts, which are in certain cafes, trebled.

† The perfon who buys the article which is to be the fubject of information, is not the informer, but is employed by the informer as a witnefs, and is generally paid at fo much for each information laid, whether conviction follows or not. Now, if it could be proved that the witnefs had any intereft in the conviction, it would be quafhed; but it is extremely difficult to come at fufficient proof of this. They are in all fhapes, male and female; but the greater part are decently dreffed women, who are more infidious, and more difficult to be guarded againft than thofe of the other fex, as being lefs fufpected, taking their prey off their guard by engaging them in fmall-talk, and by other wiles.

Gentlemen who, from local advantages of ſituation, or from other circumſtances, find their account in diſpenſing medicines by retail, may think themſelves ſafe from the penalties of the act, ſo long as they refrain from SELLING any of the articles brought within the meaning of the act.

Then they muſt turn one half of the goods of their ſhop out of doors. They will find, that if they *keep them in their shop*, even though they do not *sell* them, they are in danger. The twelfth clauſe of the Act lays its claws on all ſuch. Thus it runs:

Clauſe XII. *And be it enacted, that no person shall utter, vend, or expose to sale, or* KEEP READY FOR SALE, *or keep for the purpose of selling by retail, any drug, herb, medicine, &c. subject to the stamp duties, unless the stamp shall be well and sufficiently pasted, &c. thereto,* PREVIOUS *to such sale, or that shall have a stamp of lower value than by this Act is directed, under the penalty of ten pounds.*

Now, how is a man, with ſuch a clauſe as this hanging over his head, to carry on his buſineſs? If, keeping a retail ſhop, he keeps compound tincture of bark, arrow root, Spaniſh juice, aqua litharg. acetat, tincture of anguſtura, any kind of troches or lozenges whatever, ſyrup of tolu, any kind of corn ſalve, or any thing that can be conſtrued into a powder that may be uſed as a dentifrice, without having a ſtamp ad valorem affixed thereto, is liable to the penalty.

There are few gentlemen of the profeſſion that have not ſome formula of their own, by which they make, and keep ready for occaſionally diſpenſing, certain compoſitions; for inſtance, pills of which they prepare a large quantity at once, to ſave the trouble of making them every time a ſingle box may be wanted. Theſe are labelled according to their qualities; tonic pills, deobſtruent pills, quaſſia pills, aperient pills, &c.; all ſuch are liable!

Now, though a man may not abſolutely intend to ſell any one of theſe articles; yet, if he keeps a retail ſhop for the ſale of other medicines, I queſtion whether he would not be conſidered as keeping thoſe above-mentioned for the purpoſe of ſale, if found in the ſhop.

I know not how this is to be aſcertained, ſo as to bring offenders againſt this clauſe to condign puniſhment. Whether inſpectors are to be appointed, who may enter our houſes at all times, and ranſack every hole and corner to find unſtamped goods; or whether our own ſhopmen, apprentices, and ſervants are to be inveſted with full powers to hold the rod of correction over us. The reward for informing is certainly not inconſiderable, the ways by which the penalty may be incurred are many; and any man's ſhopman or apprentice, who owes his his mafter a grudge, has it in his power to ruin a whole family.

Neither do I know how, in fome cafes, the ftamp is to be affixed. Some of my cuftomers are in the habit of calling for an ounce of Huxham's tincture of bark, and drinking it in the fhop, out of the graduated glafs that I meafure it in. Now, I would fain know of the ingenious framer of the act, how *I* am to act in fuch a cafe? As my cuftomer refufes to have a phial, am I to pafte the ftamp on the graduated glafs, while he is drinking his dram? or, am I to infift on his fwallowing ftamp and all?

The fourth claufe and the nineteenth are at variance with each other. The fourth claufe enacts, that the duties are not to extend to articles mentioned in the book of rates, nor to *unmixed* drugs fold by a regular furgeon, apothecary, or druggift.

The nineteenth claufe enacts, that every herb, drug, or preparation whatever, that ever was, is now, or ever fhall be, recommended by any printed or written paper, as beneficial for curing the ailments, aches, and diftempers that human flefh is heir to, muft be fold with ftamps. So, though the fourth claufe clearly exempts Spanifh juice, refined liquorice, and Turkey rhubarb, which are certainly unmixed drugs, and arrow root, which is no drug at all, but an article of food; the ninteenth claufe contradicts this, inafmuch as it ordains, that every article fet forth in the Schedule fhall be fubject to the duty, and fold with ftamps; and thefe four, as well as other articles in the fame predicament, are expressly named in the Schedule!

How are fuch contradictions to be reconciled?

Surely, if every herb, drug, or compofition, that "ever was, "is now, or ever hereafter fhall be, by any public notice or "advertifement, recommended as fpecifics, or as beneficial for "the prevention, cure, or relief of any diftemper incident to, "or affecting the human body," is to be conftrued to come within the meaning of the act, there is fcarcely an article of the materia medica that is not liable to a ftamp duty! A book written to make known the virtues and properties of any medicinal herb, &c. is the moft *public notice* that can be given. A treatife is *an advertisement*. In this point of view I fhould deem it almoft dangerous to fell chamomile flowers without a ftamp; for many have been the treatifes written thereon. Dr. Kentifh, and my friend George Wilkinfon of Sunderland, have publifhed advertifements, i. e. treatifes on the *beneficial* effects of common oil of turpentine in *burns and scalds:* So that, if I underftand the meaning of the act aright, we cannot apply a rag dipped in oil of turpentine to a fcalded foot, without pafting a three halfpenny ftamp on it; (the difficulty will be, how to make the paſte to ſtick, or how faſten the ſtamp on, ſo as to comply ſtrictly with the *very rigid injunctions* concerning *pasting*, *threading*, *sticking*, *fastening*, *and affixing*, that are laid down in the eleventh clauſe of the act.

Dr. Saunders has written on the red bark; another gentleman has recommended the yellow bark in a treatiſe; Mr. James and Mr. Wilkinſon celebrate the broad-leafed willow bark; and the reſpectable Mr. Brande, apothecary to the Queen, the anguſtura bark.

If the treatiſes on theſe ſubjects are not *public notices*, I do not know what the word "public notice" means; and if it be granted that they are ſo, the framer of the act had as good a right to include them and every other ſimple drug that ever was written on, in his Schedule, equally as well as Turkey rhubarb or Indian arrow root.

The *tincture* of Anguſtura is indeed ſentenced, as well as Huxham's tincture, and tincture of rhubarb *, to bear the diſgraceful ſtamp, originally intended to attach only to the ſecret preparations of a noſtrum-monger; and the Stizolobium or Dolichos pruriens, that has been with ſome credit making its way into the world theſe twenty years, ſanctioned by the approbation of many medical characters of the firſt reſpectability, who have recommended, preſcribed, and exhibited it, muſt now go forth into public, wearing on its back the opprobrious livery of Empiriciſm.

That truly eminent friend to mankind, Dr. Carmichael Smyth, has deſervedly been rewarded by the Legiſlature of this country, for the important ſervices rendered to it, by his diſcovery and communication of a method of preventing contagion by means of nitrous fumigation: and yet I queſtion whether an apothecary, who ſhould make up parcels of ingredients for making the nitrous fumigation, and deliver with them a printed paper of inſtructions, ſhewing how they are to be uſed, and what diſeaſes ſuch fumigation is conducive to prevent, would not render himſelf liable to a confiſcation of part of his property, for ſelling them without ſtamps, and without having a quack-medicine licence!

A more effectual way to check the diſcoveries of ingenious medical men could not be deviſed, than to oblige thoſe who exert their endeavours to benefit the community, by preparing and diſpenſing, (as in the caſe of Huxham's tincture of bark) that which an author or writer may recommend and lay open to all

* Of twenty medical men, met together to diſcuſs the act, ten were of opinion that tincture of DITTO (in the Schedule) referred only to *tincture of Turkey rhubarb*, while the other ten contended, that tincture of rhubarb, *generally*, was underſtood.

all the world, to degrade themfelves by adopting the humiliating fituation and infignia of a vender of quack noftrums.

I have been led much farther into the fubject than I at firft intended, and have dwelt fo much on the *Disease*, that it is now high time to haften to the Method of Cure.

The Medicine Act, now in force, expires on the firft of next September; and from and after that day, the new Act takes place; licences under the old act are then no longer in force, but new licences muft be taken out.*

The penalty for vending, without ftamps, articles within the meaning of the act, being double the fum impofed by the old act, and quadrupled for felling without a licence, it will be fafeft for all who think themfelves in danger, to facrifice forty fhillings, as they will find it fo very difficult to efcape incurring the pains and penalties of the law by fome act of inadvertence or other, of themfelves or their domeftics, however cautious and circumfpect they may be.

The act, as before obferved, takes place from and after the firft of September; but as the new Parliament are not expected to meet until fome time after, we cannot petition for a remedy until the Parliament fhall have met for tranfacting bufinefs; and much mifchief may be done in the interim.

The only thing that can *now* be done, is, to endeavour to obtain, by lawful means, a fufpenfion or indemnification from all fuch profecutions as may be brought in confequence of the contradictory claufes contained in the new act, againft fuch perfons as fhall have vended various articles agreeably to the letter and fpirit of the fourth and fifth enacting claufes, notwithftanding fuch articles are enumerated in the Schedule as liable to the ftamp duties.

For this purpofe, it is intended to prefent a refpectful Memorial to the Lords of the Treafury, praying for that relief; and a very numerous body of Surgeons, Apothecaries, Druggifts, and Chemifts, with fome other gentlemen, not of the medical profeffion, but materially affected in their bufinefs by the operations of the act, who held a meeting at the New London Tavern, on the 5th day of July, have appointed a Committee, whom they have empowered to draw up faid Memorial; to call general meetings, when found expedient; and to take fuch other meafures as may be, from time to time, thought neceffary for the fecurity of thofe affected or aggrieved by the operations of the act.

Next,

* The coft of a licence in the metropolis and within the limits of the two-penny poft, is forty fhillings; in any city, borough, or town corporate; alfo in Manchefter, Sheffield, or Birmingham, ten fhillings; elfewhere, five fhillings.

Next, I would beg leave to recommend, that every Practitioner in Pharmacy, who has accefs to any of the members of the new Parliament, and whofe habits of intimacy with fuch member will allow of the meafure, fhould take the trouble of fetting forth to him fuch parts of the act as are moft exceptionable, and bear hardeft on the fubject; and explain to him in what manner it is poffible very innocent and unintentionally-offending individuals* may be totally ruined; with a view that the members of the Legiflature, both Lords and Commons, may be made mafters of the fubject, when a Petition for relief comes before the Houfe.

It will be indifpenfibly neceffary, that the Practitioners in Pharmacy, and others connected with the act, fhould call meetings among themfelves in every county and town, and enter into fubfcriptions for purpofes hereafter to be mentioned; but the grand object fhould be, the petitioning Parliament for relief. Whether that relief fhould be by a MODIFICATION, or REPEAL of the act, is not for me to fay.

But, in cafe the Memorial, intended to be refpectfully prefented to the Lords of the Treafury for a fufpenfion or indemnification from profecutions under the new act, until fuch time as the decifion of Parliament, in confequence of a Petition for relief fhall be known, fhould fail of fuccefs, or in cafe there fhould be any delay of time before their Lordfhips' pleafure fhould be made known; as, in either of thefe cafes, numberlefs harraffing and vexatious informations may be brought againft perfons wholly ignorant of the fcope of the new act, it will be proper that the fubfcriptions entered into, in every diftrict, be liberal, and that all parties concerned, fhould unite in defence of each other, for the purpofe of defraying the expences, and of defending Qui-tam informations brought againft Subfcribers, who are led, unintentionally, into an infringement of the act, and againft whom, particular art and cunning can be proved to have been ufed to entrap them.

But here it is to be cautioufly obferved, that in every Affociation which may be fet on foot for the purpofes of mutual defence, there ought to be one ftrong, firmly-eftablifhed, coercive rule, never to be departed from on any account whatever, namely, that the Committees, appointed by the refpective bodies

* There are feveral articles fpecified in the Schedule, that grocers, confectioners, perfumers, and thofe country fhop-keepers who traffic in a very great variety of goods, muft indifpenfibly deal in. All thefe defcriptions of traders therefore are affected by the new medicine act, equally as well as thofe who fell or difpense medicines. Even the poor widow who keeps a gingerbread ftall, and fells a halfpenny lollypop, will, if fhe fells it in the *name*, or in the *shape*, of a LOZENGE, have all the terrors of the act to dread.

dies for managing their businefs, fhall be WHOLLY RESTRICTED FROM DEFENDING ANY SUBSCRIBER WHO SHALL APPEAR TO OFFEND AGAINST THE ACT, EITHER THROUGH GROSS NEGLIGENCE, OR A DESIGN TO EVADE THE DUTIES. And, that no perfon may plead ignorance, it will be expedient to appropriate a portion of the fubfcription money in printing an Abridgment of the Act of Parliament, or of fuch Claufes of faid Act as are indifpenfibly neceffary to be made known, together with fuch Comments or Obfervations as may explain and point out to the meaneft capacity, what cautions are to be obferved, fo as not to offend againft the act, together with *the whole* of the Schedule, recommending it to every perfon concerned, to make a *regular* alphabetical lift of fuch articles mentioned in the Schedule, as he or fhe may deal in, which lift fhould be pafted, or affixed permanently, in fome convenient part of the fhop; and in large fhops there fhould be feveral lifts hung up in different places, to be referred to in a moment when an article is called for that the fhopkeeper is in doubt about, with a table of the ftamp duty attached to every article; for among people unaccuftomed to this businefs of affixing ftamps upon their goods, there will be greater confufion, and more informations laid for putting on wrong ftamps, than for felling with no ftamps at all.

Thefe cautionary papers ought to be printed at the expence of a public fund, and the managing Committee fhould take care that one of them fhould be delivered to every furgeon, apothecary, druggift, chemift, perfumer, grocer, confectioner, and every perfon whatever, known or fuppofed to vend any article or articles within the meaning of the act, whether fubfcribers to the fund or not; for which a certain price fhould be demanded and paid. The Subfcribers might be allowed to have it at a price juft fufficient to defray the expences of paper, printing, and circulating. Non-fubfcribers fhould pay more; and no one will grudge to pay a fmall fum for that which may eventually be the means of faving the purchafer from a penalty of fifteen or twenty pounds the very next day, or perhaps from a workhoufe.

What other fteps it may be neceffary to take, I fhall not prefume to dictate; at all events, the fafeft way will be for thofe who think themfelves affected, to take out licences, and be particularly careful to attend ftrictly to the letter of the law.

Whether I am perfectly correct in my conceptions refpecting the operations of the act in certain cafes, I do not know. In *fome* things I may be miftaken, and I wifh it may fo turn out; but I find that, generally, other perfons are of the fame opinion with refpect to the meaning of the different claufes as myfelf.

Whether

Whether a modification of the act, or a repeal be prayed for, it will be neceffary, previoufly thereto, for thofe who petition, to take certain matters of importance into confideration, that the revenue may not fuffer lofs by the indulgence. I have an opinion on that fubject; but as I have already exceeded bounds, and as it is a matter that will keep cool, I fhall not prefume now to offer it.

And now, Sir, I have to apologize for taking up fo many pages of your valuable publication, and for fo long trefpaffing on the time of your Readers. The Irifhman's poftfcript, "Excufe hafte, for I have not time to make my letter fhorter," literally applies to me in the prefent inftance. It is true, I had read in the newfpapers, that a *new* Medicine Act had paffed; but as I deal not in noftrums, and hold all forts of Quackery in deteftation; like many others of my brethren, I fuppofed it had nothing to do with me, nor I with it, of courfe I never read it, nor knew any thing of its tendency until the third day of this month; and the fhort fpace of time between that day and the time limited by your publifher for fending in Communications, fo as to be early enough to appear in the enfuing month, would not admit of my fparing fo much time from a variety of profeffional and other avocations, as would be required for a careful abridgment of this letter, which I have certainly fpun out to a length far beyond what I at firft imagined I fhould have gone to, and which I moft undoubtedly fhould have curtailed if time had permitted.

I truft, that the importance of the fubject to Practitioners in Pharmacy, and the circumftance of its being a matter that cannot be made known too foon, will plead in my behalf for its being fent into the world with many crudities and imperfections. I have very little doubt but that had I printed it in a pamphlet, efpecially in the prefent modern way, "on a large type, and with a rivulet of print running through a meadow of margin," I could have fpun it out to a tolerable fized two fhilling pamphlet, and I dare fay it would have fold well, and brought me fome emolument; but the welfare of my brethren of the profeffion, and the neceffity there was for warning them of their danger without lofs of time, was paramount with me to every confideration for felf. I confidered too, that fuch a book, even if well advertifed, might poffibly not find a circulation much beyond the environs of the metropolis, but that the very extenfive circulation of the Medical Journal throughout all parts of England and Scotland, would give the moft general and early notice to Medical Practitioners, even in the remoteft parts of Cornwall and Caledonia, of what is going forward, and what concerns them to know; with this advantage, advantage, that they will now have that information gratuitoufly, which otherwife they muft have paid for. If by it, I fhould be the means of faving any practitioner from the heavy loffes which the incurring of penalties under this act might occafion; to hear that through the means of fuch information, fo conveyed, the party was faved from incurring fuch penalties, will afford me far greater gratification than could any pecuniary emolument arifing from the fale of a book.

I truft, Sir, I have deferved well of my brethren of the Medical Profeffion. It is with an endeavour fo to do, I have now trefpaffed on you; and I hope and believe, I have deferved well of my country; at any rate, I have the vanity to think, that a great many more copies of the act of parliament will be purchafed, a great many more licences taken out, and a great many more ftamps fold, in confequence of my having written this, than if I had not written it; and of courfe the national revenue will profit by my labours, as well as individuals.

*Aylesbury Street, Clerkenwell, July* 12, 1802.

